# The down-regulation of SLC7A11 enhances ROS induced P-gp over-expression and drug resistance in MCF-7 breast cancer cells

**DOI:** 10.1038/s41598-017-03881-9

**Published:** 2017-06-19

**Authors:** Chun Ge, Bei Cao, Dong Feng, Fang Zhou, Jingwei Zhang, Na Yang, Siqi Feng, Guangji Wang, Jiye Aa

**Affiliations:** 1 Laboratory of Metabolomics, Key Laboratory of Drug Metabolism and Pharmacokinetics, China Pharmaceutical University, Nanjing, 210009 China; 2 Nanjing Drum Tower Hospital, The Affiliated Hospital of Nanjing University Medical School, Nanjing, 210009 China

## Abstract

Adriamycin (ADR) induces the over-expression of P-glycoprotein (P-gp) and multiple drug resistance in breast cancer cells. However, the biochemical process and underlying mechanisms are not clear. Our previous study revealed that ADR increased reactive oxygen species (ROS) generation and decreased glutathione (GSH) biosynthesis, while N-acetylcysteine, the ROS scavenger, reversed the over-expression of P-gp. The present study showed that ADR inhibited the influx of cystine (the source material of GSH) and the activity of the SLC7A11 transporter (in charge of cystine uptake) in MCF-7 cells. For the first time, we showed that the down-regulation/silence of SLC7A11, or cystine deprivation, or enhanced ROS exposure significantly increased P-gp expression in MCF-7 cells. The down-regulation of SLC7A11 markedly enhanced ROS induced P-gp over-expression and drug resistance in MCF-7 cells; a combination of either an inhibited/silenced SLC7A11 or cystine deprivation and increased ROS dramatically promoted P-gp expression, which could be reversed by N-acetylcysteine. In contrast, the over-expression of SLC7A11, or supplementation with sufficiently cystine, or treatment with N-acetylcysteine significantly decreased P-gp expression and activity. It was suggested that ROS and SLC7A11/cystine were the two relevant factors responsible for the expression and function of P-gp, and that SLC7A11 might be a potential target modulating ADR resistance.

## Introduction

Chemotherapy is one of the most effective treatments in current breast cancer therapy. Since the introduction of chemotherapy and earlier diagnoses in the middle 1990s, breast cancer-induced morbidity and mortality have been significantly reduced, and the lifespan of breast cancer patients has been prolonged^1^. However, the development of drug resistance eventually becomes a great challenge to the successful chemotherapy of breast cancer. It is estimated that a significant number of breast cancer patients (up to 50%) are not responsive to current chemotherapeutic regimens^2^. Resistance to a single antitumour drug tends to trigger the development of pleiotropic resistance to a wide variety of structurally and functionally independent anticancer agents, and this phenomenon is called multidrug resistance (MDR). It has been established that membrane proteins, such as the multidrug resistance protein (MRP) and the breast cancer resistance protein (BCRP) of the ATP binding cassette (ABC) transporter family that encodes efflux pumps, play key roles in the development of the multidrug resistance phenotype. The over expression of these transporters has frequently been observed in many types of human malignancies that respond poorly to chemotherapeutic agents.

As a frontline anti-tumour drug, Adriamycin (ADR) is a DNA intercalating agent and one of the most active agents against breast cancer^3, 4^. Unfortunately, like many other chemotherapeutic agents, the continuous administration of ADR causes drug resistance so that the therapeutic efficacy dramatically declines^5^. Clinical data have confirmed that both chemotherapy sensitivity and patient survival were negatively correlated with P-glycoprotein (P-gp) expression. Current evidence has suggested that ADR resistance is deeply involved in the induction of highly expressed P-gp, which, in turn, enhances the efflux of ADR from inside cells^6^. Additionally, the breast cancer cell line MCF-7 can be induced by fairly low exposure to ADR *in vitro* to become the stable ADR-resistance cell line MCF-7R, with the distinct biological characteristic of hundreds of times higher P-gp expression. To date, it remains elusive which factor plays a crucial role in initiating the over-expression of P-gp, although genomic and proteomic studies have suggested that potential factors such as cyclin, apoptin, microRNA-451, and Anxa2 are involved at the transcriptional and posttranscriptional levels^7–14^.

Recently, accumulated evidence has demonstrated that, when cells are treated with antitumour drugs, redox signals are activated and affect processes such as apoptosis, metastasis, and the inflammatory response, leading to attenuated efficacy. It was generally accepted that redox signals modulate the transporters of membrane proteins via mechanisms that include (a) conformational changes of the transporters, (b) regulation of the biosynthesis cofactors required for the transporter’s function, (c) regulation of the expression of transporters at the transcriptional, posttranscriptional, and epigenetic levels, and (d) amplification of the copy number of the genes encoding these transporters. However, genetic and proteomic studies did not identify the direct factors contributing to the generation and homeostasis of reactive oxygen species (ROS), and conflicting opinions emerged regarding the relationship and mechanism between redox signaling and multidrug resistance in cancer cells^15–19^. Our previous study of metabolomics revealed that ADR switched metabolic pathways in MCF-7S cells, and the ADR-resistant cell line MCF-7R appeared to demonstrate a distinctly altered metabolic pattern from that of the sensitive cell line MCF-7S. For example, we found that ADR promoted notable metabolism reprogramming, including markedly disturbed biosynthesis of proteins, purines, pyrimidines, glutathione and glycolysis, whilst enhancing the glycerol metabolism of MCF-7S cells^20^. The elevated glycerol metabolism and down-regulated glutathione biosynthesis suggested an increased ROS generation and a weakened ability to balance ROS, respectively. Oxidative stress and elevated ROS usually enhance the production of reduced glutathione (GSH) via the up-regulation of the expression of γ-glutamylcysteine synthetase (γ-GCS), the rate limiting enzyme for the biosynthesis of GSH^21^. Therefore, the obvious contradiction between the increased ROS and decreased GSH induced by ADR greatly raised our interest in the underlying mechanism contributing to the up-regulation of P-gp.

Thus, the present study was designed to clarify the effect of ADR on the generation and homeostasis of ROS (e.g., the synthesis of GSH), and redox regulation of P-gp over-expression induced by ADR. As a crucially important node of the cellular antioxidant system, SLC7A11 encodes for a subunit of the xCT cystine/glutamate amino acid bilateral transport system, and cystine represents the synthesis of GSH^22–24^. Based on our previous results^20^, we fully investigated the role of SLC7A11 and cystine in the over-expression of P-gp in the current study. Moreover, to exclude the possible direct activity of ADR on P-gp, the effect of the direct addition of ROS agents and the direct modulation on SLC7A11 or cystine, alone or in combination, were evaluated, although a potential correlation between ROS and P-gp has been reported^25–27^. Our goal was to examine the specific role of elevated ROS coupled with a decreased anti-oxidative capacity in the ADR-induced over-expression of P-gp.

## Results

### The differential impact of ADR on GSH/GSSG ratio, the influx of cystine and the expression of SLC7A11 in MCF-7S and MCF-7R cells

**Figure 1:**
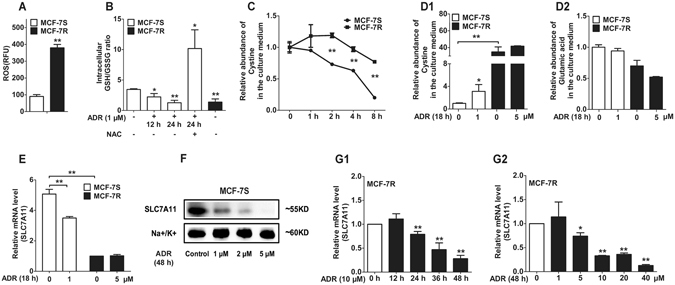
The differential impact of ADR on GSH/GSSG ratio, the influx of cystine and the expression of SLC7A11 in MCF-7S and MCF-7R cells. (**A**) The baseline ROS level in MCF-7R cells was remarkably higher than in MCF-7S cells. (**B**) ADR reduced the intracellular GSH/GSSG ratio in MCF-7S cells to a comparable level to that of MCF-7R cells, which was reversed by NAC (10 mM, 30 min of pretreatment). (**C**) The utilization of cystine was much higher in the culture medium of MCF-7S cells than that of MCF-7R cells, although both groups were treated with the same initial concentration (1.0 mM). (**D**) ADR inhibited the influx of cystine and the efflux of glutamic acid (substrates of SLC7A11) in MCF-7S (1 μM) and MCF-7R (5 μM) cells. (**E**) The SLC7A11 mRNA level in MCF-7R cells was intrinsically lower than in MCF-7S cells. Moreover, ADR markedly inhibited SLC7A11 mRNA expression in MCF-7S cells (1 μM), but showed a marginal effect on MCF-7R cells (5 μM). (**F**) ADR down-regulated SLC7A11 protein expression in MCF-7S cells. (**G**) The inhibitiory effect of ADR on SLC7A11 mRNA expression showed time-dependence and concentration-dependence in MCF-7R cells. All of the experiments were conducted in triplicate, and data with error bars are presented as the mean ± SD (n = 3). *p < 0.05; **p < 0.01 vs. control.

It was previously shown that ADR promoted the generation of ROS in MCF-7S cells^20^. In additon, ADR significantly increased P-gp expression dose-dependently and time-dependently, while the ROS scavenger N-acetylcysteine (NAC) exhibited a distinct antagonistic effect, suggesting that ADR resistance was involved in aggravated oxidative stress^20^. In the present study, we found that the level of ROS in ADR-induced MCF-7R cells was approximately 4 times that in MCF-7S cells (Fig. 1A), and the baseline GSH (reduced glutathione)/GSSG (oxidized glutathione) ratio of MCF-7R cells was lower than that of MCF-7S cells, indicating a poor capacity for the removal of ROS^28, 29^, in concordance with previous results (Fig. 1B). In addition, ADR treatment decreased the intracellular GSH/GSSG value in MCF-7S cells, which was significantly reversed by NAC (Fig. 1B).

To monitor cystine (the source material for the synthesis of GSH) uptake, extracellular cystine of both MCF-7S and MCF-7R cells was detected by GC/MS, with the same initial concentration of cystine (1.0 mM) in the culture medium. We observed that extracellular cystine was much higher in MCF-7R cells than in MCF-7S cells over time, suggesting a much faster influx/utilization of cystine in MCF-7S cells than in MCF-7R cells (Fig. 1C). In fact, SLC7A11 controls not only the uptake of cystine but also the efflux of glutamic acid^22–24^. Our data showed that ADR intervention reduced the influx of cystine into cells, and slightly reduced the efflux of glutamate out of cells so that more cystine and less glutamate were present in the culture medium (Fig. 1D), indicating that ADR significantly inhibited the function of SLC7A11. Further examination of SLC7A11 at the mRNA level revealed that its expression in MCF-7S cells was approximately 5 times that in MCF-7R cells, the ADR-induced cell line (Fig. 1E). Moreover, a significant inhibition of SLC7A11 was observed in MCF-7S cells after exposure to ADR (Fig. 1E and F). For MCF-7R cells, a longer exposure at higher levels also significantly inhibited SLC7A11 (Fig. 1G), although MCF-7R cells did not respond to ADR at the same level as for MCF-7S cells. Together with our previous data^20^, the above results revealed that, in addition to P-gp over-expression, ADR increased ROS production and inhibited SLC7A11 function and cystine uptake, suggesting an attenuated capacity for anti-oxidative stress with a decreased GSH/GSSG ratio.

### Effects of ROS agents on P-gp expression in MCF-7S cells

**Figure 2:**
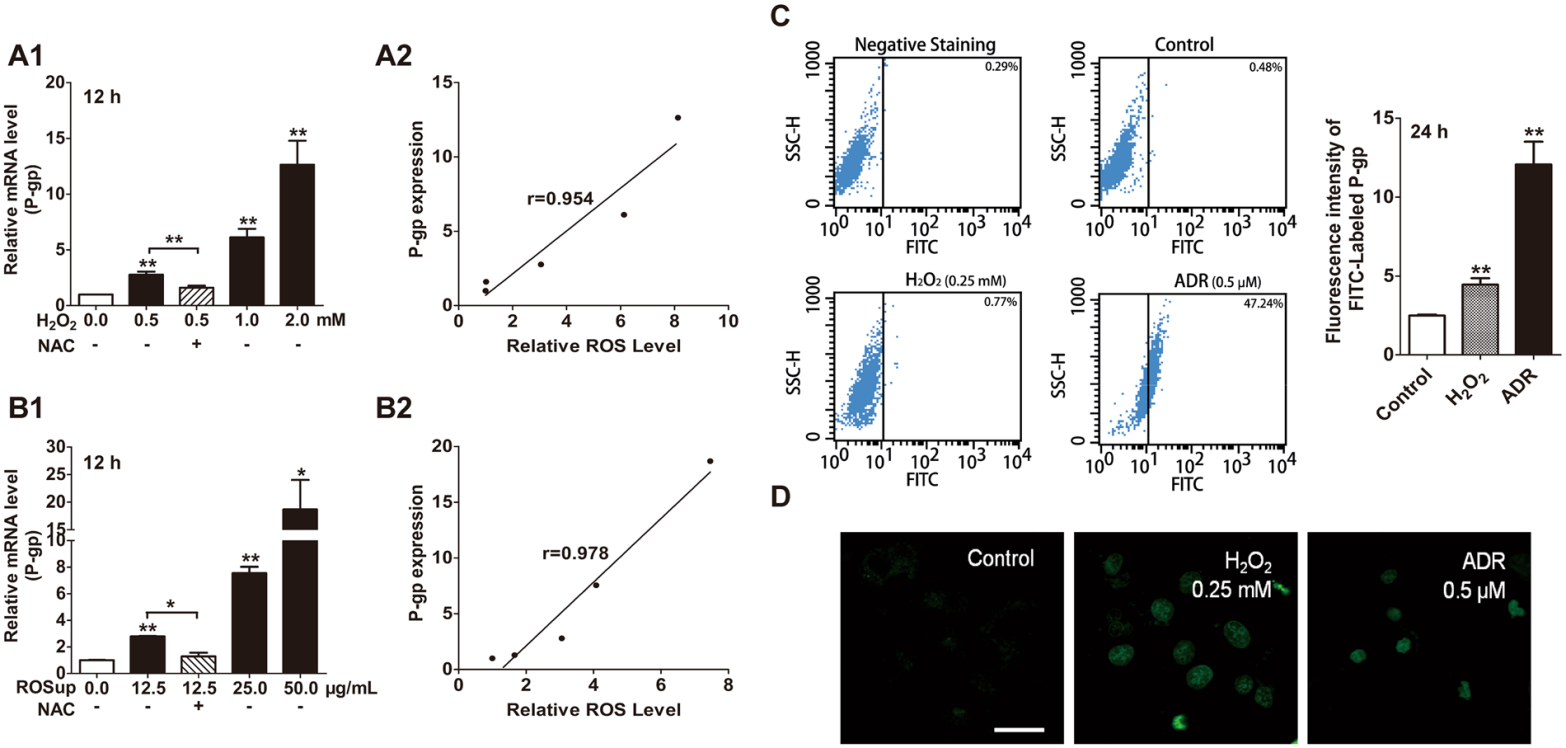
Effects of ROS agents on P-gp expression in MCF-7S cells. (**A**) H_2_O_2_ increased P-gp mRNA expression, which could be reversed by NAC (10 mM, 30 min of pretreatment). (**B**) ROSup increased P-gp mRNA expression, which could be reversed by NAC (10 mM, 30 min of pretreatment). (**C**) H_2_O_2_ (0.25 mM, 24 h) or ADR (0.5 μM, 24 h) enhanced P-gp protein expression, as demonstrated via by flow cytometry analysis and quantified according to mean fluorescence intensity. (**D**) H_2_O_2_ (0.25 mM, 24 h) or ADR (0.5 μM, 24 h) enhanced P-gp protein expression, as shown with laser confocal scanning using an FITC-conjugated anti-P-gp antibody. Scale bar: 20 µm. All of the experiments were conducted in triplicate, and data with error bars are presented as the mean ± SD (n = 3). *p < 0.05; **p < 0.01 vs. control.

The level of ROS in MCF-7R cells, a stable cell line induced by continuous induction of ADR, has been shown to be approximately 4 times that in MCF-7S cells (Fig. 1A). Moreover, ADR significantly elevated the ROS level and P-gp expression, which could be reversed by NAC^20^, suggesting a distinct correlation between the intracellular ROS level and P-gp over-expression. To exclude other potentially latent factors involved in P-gp over-expression and to explore the direct correlation between ROS and P-gp, MCF-7S cells were exposed to two typical ROS agents. It was confirmed that the ROS agents, H_2_O_2_ and ROSup dose-dependently produced a large amount of ROS (Supplementary Fig. S1). Thus, we found that as the ROS production increased, the mRNA expression of P-gp was higher, which could be efficiently reversed by NAC, indicating a direct effect of ROS on the elevation of P-gp (Fig. 2A and B). Consistent results were also achieved by immunofluorescence analysis, with flow cytometry (Fig. 2C) and laser confocal scanning (Fig. 2D). Nevertheless, the highest level of ROSup greatly promoted the expression of P-gp as much as 19.6 fold (Fig. 2B1), indicating that an extensive or sustained exposure and excessive ROS remarkably enhanced P-gp expression. These results strongly suggested that ROS was an initial factor contributing to the over-expression of P-gp.

### Modulatory effects of SLC7A11 on P-gp expression and function

**Figure 3:**
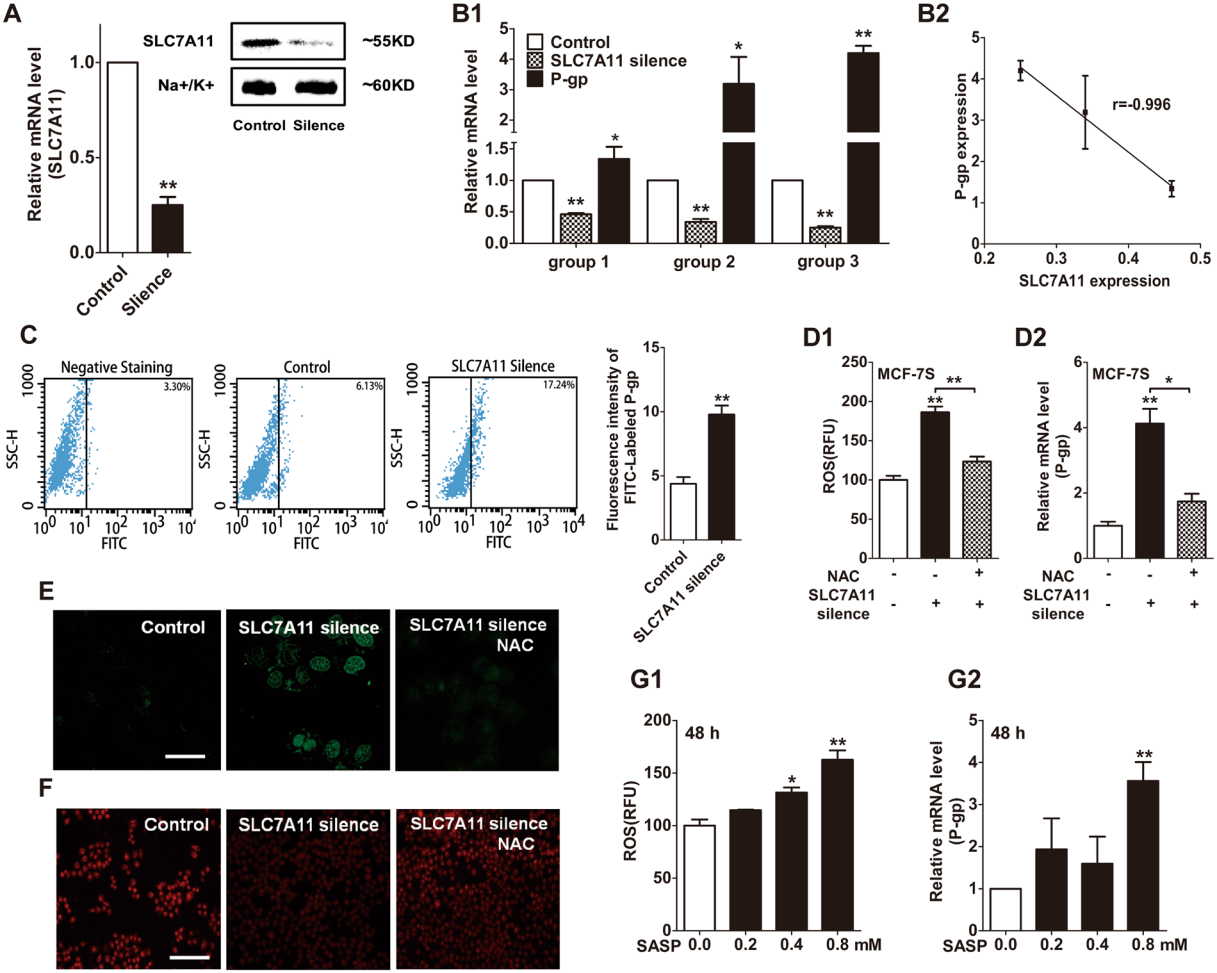
Down-regulatory effects of SLC7A11 on P-gp expression in MCF-7S cells. (**A**) SLC7A11 mRNA and protein levels were efficiently reduced by siRNA. (**B**) SLC7A11-siRNA increased P-gp mRNA expression, in a negatively dependent way. (**C**) SLC7A11 silence stimulated P-gp expression, shown by immunofluorescence analysis, with flow cytometry. (**D**) SLC7A11 silence increased the intracellular ROS level and P-gp mRNA expression, which could be reversed by NAC (10 mM, 24 h). (**E**) SLC7A11 silence enhanced P-gp protein expression, which could be reversed by NAC (10 mM, 24 h), as shown with laser confocal scanning analysis. Scale bar: 20 µm. (**F**) SLC7A11 silencing enhanced the function of P-gp, which could be reversed by NAC (10 mM, 24 h), and Rho 123 was identified as a typical substrate of P-gp. Scale bar: 100 µm. (**G**) Treatment with an SLC7A11 inhibitor, sulfasalazine, increased ROS levels and P-gp mRNA expression. All of the experiments were conducted in triplicate, and data with error bars are presented as the mean ± SD (n = 3). *p < 0.05; **p < 0.01 vs. control.

It is well known that, SLC7A11 serves as the major means of increasing intracellular cysteine and accelerating the production of GSH^22–24^. It was shown that SLC7A11 expression in MCF-7R cells was much lower than that in MCF-7S cells, and ADR inhibited SLC7A11 expression in MCF-7S and MCF-7R cells (Fig. 1E–G). We further examined the regulatory effect of SLC7A11 on the expression and function of P-gp with siRNA (Fig. 3A). We found that when the SLC7A11 expression level was decreased by 25%, 34%, 46% via siRNA silencing, in response, P-gp mRNA expression was increased by 1.34, 3.19, and 4.20 times, respectively, compared with controls (Fig. 3B1). Additionally, there was a negative correlation between the mRNA level of SLC7A11 and P-gp (Fig. 3B2). It was also shown that the down-regulation of SLC7A11 enhanced P-gp protein expression, which was confirmed by immunofluorescence analysis with flow cytometry (Fig. 3C) and laser confocal scanning (Fig. 3E). Moreover, fluorescence microscopy analysis of intracellular Rhodamine 123 (Rho 123, the fluorescent substrate of P-gp) showed that the function of P-gp was enhanced after SLC7A11 was silenced, indicated by the increased efflux of Rho 123 (Fig. 3F). The up-regulation of ROS and P-gp induced by SLC7A11 silencing could be reversed by treatment with the ROS scavenger NAC (Fig. 3D–F). In addition, an SLC7A11 inhibitor, sulfasalazine^30, 31^ increased intracellular ROS production and P-gp expression (Fig. 3G). These findings suggested that ROS was the key intermediate factor in the regulation of P-gp expression by SLC7A11.

**Figure 4:**
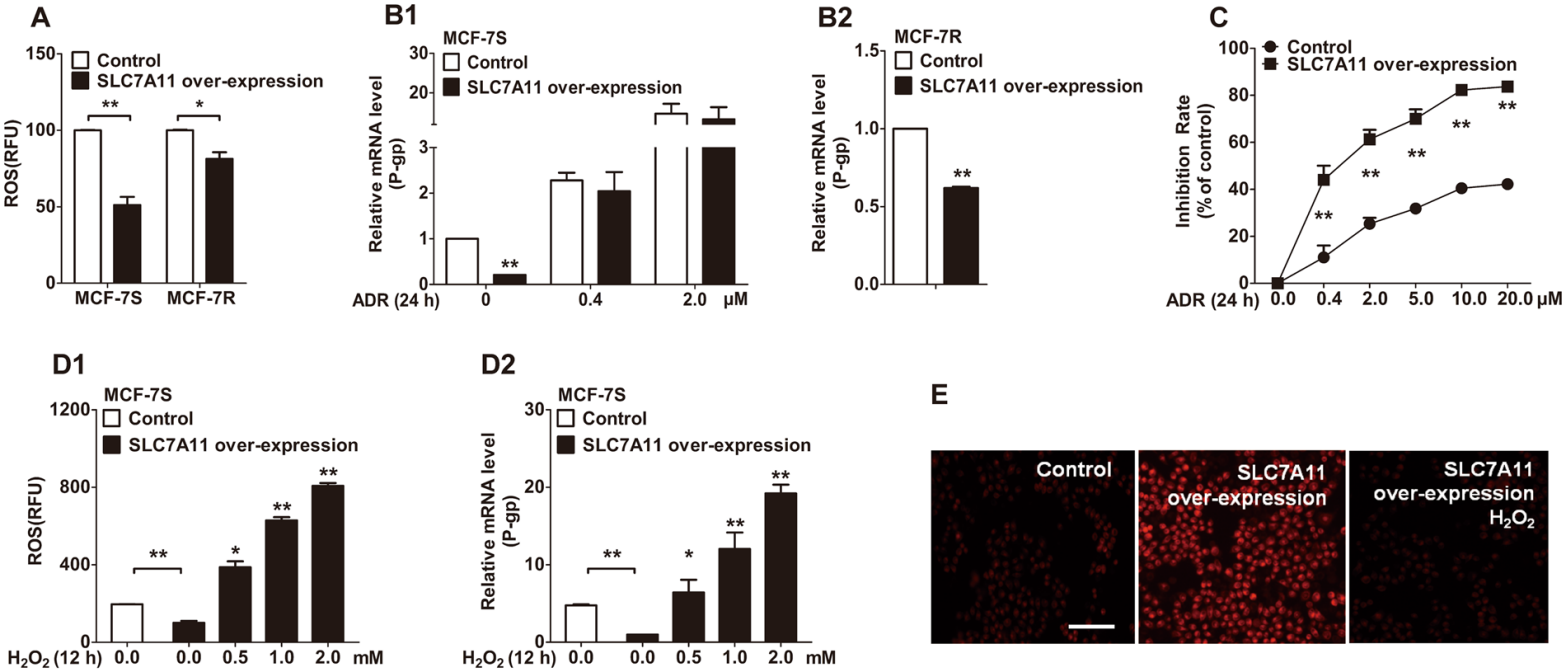
Effect of SLC7A11 over-expression on P-gp expression in MCF-7S and MCF-7R cells. (**A**) SLC7A11 over-expression reduced the ROS level in MCF-7S and MCF-7R cells. (**B**) SLC7A11 over-expression inhibited P-gp mRNA expression in MCF-7S and MCF-7R cells. Furthermore, MCF-7S cells that over-expressed SLC7A11 were more sensitive to ADR, compared to MCF-7S cells that has been exposed to a control vector. (**C**) SLC7A11 over-expression increased the cytotoxic sensitivity of ADR on MCF-7S cells. (**D**) SLC7A11 over-expression reduced ROS levels and P-gp mRNA expression in MCF-7S cells, which could be reversed by H_2_O_2_. (**E**) SLC7A11 over-expression decreased the function of P-gp, which could be reversed by H_2_O_2_ (0.25 mM, 24 h), as indicated by intracellular Rho 123 accumulation in MCF-7S cells. Scale bar: 100 μm. All of the experiments were conducted in triplicate, and data with error bars are presented as the mean ± SD (n = 3). *p < 0.05; **p < 0.01 vs. control.

By means of SLC7A11 plasmid transfection into MCF-7S and MCF-7R cells, we established two cell lines that stably and highly expressed SLC7A11 which was validated at the gene, protein and function levels (Supplementary Fig. S2). As SLC7A11 function strengthened, more extracellular cystine was taken up and more intracellular glutamate was excreted (Supplementary Fig. S2C and D), indicating the successful construction of cell lines that expressed high levels of SLC7A11. The over-expression of SLC7A11 led to significantly lower ROS levels and P-gp expression in both MCF-7S and MCF-7R cells (Fig. 4A and B), in concordance with the decreased function of P-gp, which was indicated by the increased accumulation of intracellular Rho123 (Fig. 4E). Interestingly, ADR induced a much higher fold change in P-gp expression in SLC7A11 over-expressed MCF-7S cells (Fig. 4B1), indicating that MCF-7S cells over-expressed SLC7A11 became more sensitive to ADR. In addition, ADR appeared to be more toxic to MCF-7 cells with SLC7A11 over-expression (Fig. 4C). Treatment with the ROS agent, H_2_O_2_ could reverse the down-regulation of ROS and P-gp induced by the over-expression of SLC7A11 (Fig. 4D–E). These data strongly suggested that the down-regulation of SLC7A11 was the crucially important factor that contributed to the over-expression of P-gp, mediated by the ROS level.

### Deprivation and sufficient supplementation of cystine positively or negatively affected P-gp expression in MCF-7S and MCF-7R cells, respectively

**Figure 5:**
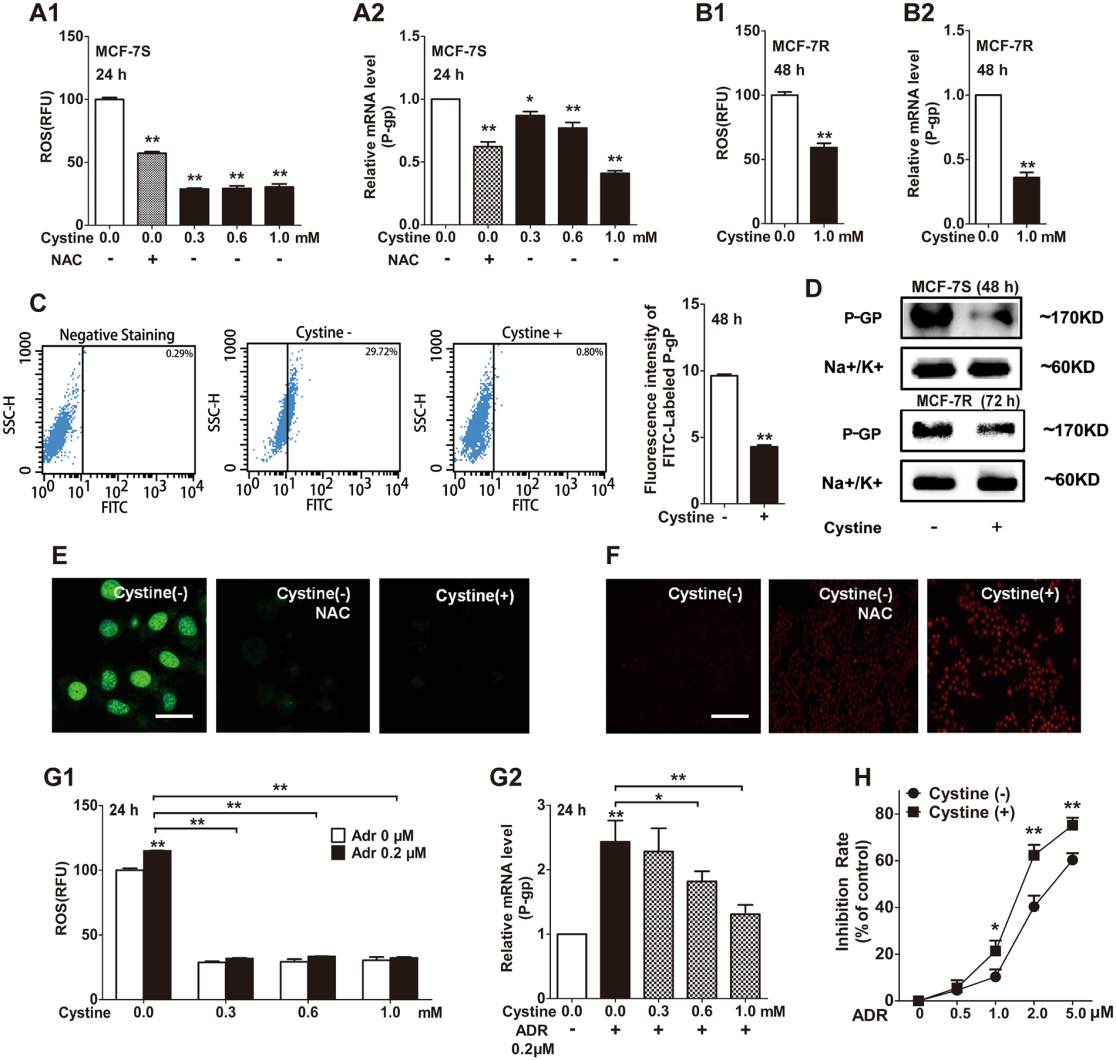
Deprivation and sufficient supplementation of cystine positively or negatively affected P-gp expression in MCF-7S and MCF-7R cells, respectively. (**A**) Additional cystine reduced ROS levels and P-gp mRNA expression in MCF-7S cells, similar to NAC (10 mM, 12 h). (**B**) Additional cystine reduced ROS levels and P-gp mRNA expression in MCF-7R cells. (**C**) Additional cystine (1.0 mM) decreased P-gp protein expression in MCF-7S cells, as shown via flow cytometry analysis and quantified according to mean fluorescence intensity. (**D**) Additional cystine (1.0 mM) reduced P-gp protein expression in MCF-7S and MCF-7R cells, as shown by Western blot. (**E**) Sufficient supplementary cystine (1.0 mM, 48 h) efficiently reduced P-gp protein expression, as did NAC (10 mM, 24 h), as shown by laser confocal scanning analysis. Scale bar: 20 μm. (**F**) Sufficient supplementary cystine (1.0 mM, 72 h) even showed greater inhibition on the function of P-gp than the effect of NAC (10 mM, 24 h), as demonstrated via intracellular Rho 123 accumulation in MCF-7S cells. Scale bar: 100 μm. (**G**) Additional cystine reversed the effects induced by ADR on MCF-7S cells, in terms of ROS levels and P-gp mRNA expression. (**H**) Additional cystine (1.0 mM, 72 h) heightened the cytotoxic sensitivity of ADR (24 h) on MCF-7S cells, as measured with the MTT assay. All experiments in this figure used cystine deprivation as a control and were conducted in triplicate. Data with error bars are presented as the mean ± SD (n = 3). *p < 0.05; **p < 0.01.

Cystine is known to be taken up through SLC7A11 and is utilized as the source material for the synthesis of antioxidant GSH^22–24^. We have shown that cystine in the culture medium is depleted more quickly in MCF-7S cells than in MCF-7R cells (Fig. 1C), and ADR decreased the influx of cystine (Fig. 1D), suggesting that cystine was involved in ADR-induced resistance. To examine the effect of cystine on ROS and P-gp levels, cystine was added or removed. Supplementary cystine significantly decreased ROS levels and P-gp expression in MCF-7S and MCF-7R cells (Fig. 5A and B). Consistent results were confirmed by flow cytometry (Fig. 5C), Western blot (Fig. 5D), laser confocal scanning microscopy (Fig. 5E) and intracellular Rho 123 analysis (Fig. 5F) in MCF-7S cells. In addition, we also found that the high levels of ROS and P-gp induced by cystine deprivation could be reversed by the ROS scavenger NAC (Fig. 5A,E and F). However, it take a longer time in MCF-7R cells (Fig. 5B and D), partly due to a slower uptake of cystine in MCF-7R cells (low expression of SLC7A11). Moreover, cystine supplementation could reverse the elevation of ROS and P-gp induced by low-dose (0.2 μM) ADR intervention (Fig. 5G), and could enhance the antitumour efficacy of ADR on MCF-7S cells (Fig. 5H). The above data suggested that cystine depletion was another factor that modulated the expression of P-gp, which primarily depended on the intracellular ROS level.

### Multiple factors of ROS generation, modulation of SLC7A11 and supplementary cystine involved in the expression and function of P-gp in MCF-7S cells

**Figure 6:**
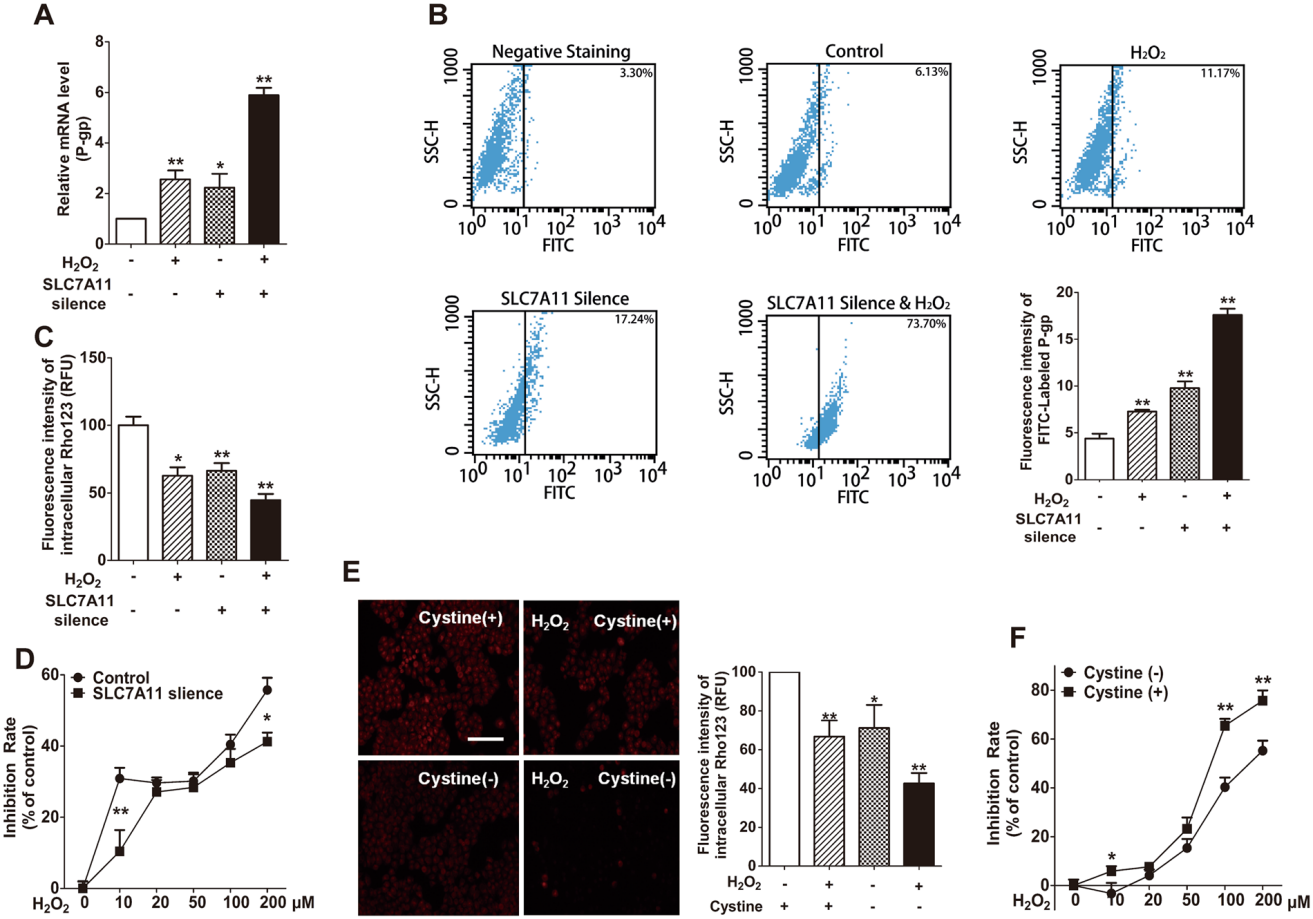
Multiple factors of ROS generation, modulation of SLC7A11 and supplementary cystine involved in the expression and function of P-gp in MCF-7S cells. (**A**) SLC7A11 silencing greatly promoted P-gp mRNA expression in the presence of H_2_O_2_ (0.5 mM, 12 h). (**B**) SLC7A11 silence greatly promoted the protein expression of P-gp in the presence of H_2_O_2_ (0.5 mM, 12 h), as shown by flow cytometry analysis. (**C**) SLC7A11 silencing significantly increased the function of P-gp in the presence of H_2_O_2_ (0.5 mM, 12 h), as indicated by decreased intracellular Rho 123 accumulation. (**D**) SLC7A11 silencing reduced the cytotoxic effect of H_2_O_2_ (24 h) on MCF-7S cells. (**E**) Cystine deprivation (72 h) significantly enhanced the function of P-gp in the presence of H_2_O_2_ (0.5 mM, 12 h), as indicated by decreased intracellular Rho 123 accumulation. Scale bar: 100 μm. (**F**) Cystine deprivation (72 h) significantly reduced the cytotoxic effect of H_2_O_2_ (24 h) on MCF-7S cells. All of the experiments were conducted in triplicate, and data with error bars are presented as the mean ± SD (n = 3). *p < 0.05; **p < 0.01 vs. control.

Our data demonstrated that the single factor of either increased ROS or inhibited SLC7A11/cystine deprivation contributed to P-gp over-expression (Figs 2–5). Therefore, we investigated the effect of the combined factors on P-gp expression and function. The results revealed that the addition of 0.5 mM H_2_O_2_ or the silencing of SLC7A11 increased P-gp expression by approximately 2-fold, which was much lower than the combination of the two factors (Fig. 6A). Fluorescence quantitation of flow cytometry and Rho123 accumulation analysis clearly showed a much higher expression and function of P-gp induced by the combination of H_2_O_2_ addition and the silencing of SLC7A11 (Fig. 6B and C). Moreover, the combination of cystine deprivation and the addition of H_2_O_2_ dramatically reduced intracellular Rho123, indicating a much stronger activity of P-gp (Fig. 6E). In addition, a weakened cytotoxicity of ADR was observed with the combination of increased ROS and inhibited SLC7A11/cystine deprivation (Fig. 6D and F), in accordance with the significant up-regulation of P-gp. These data strongly indicated that the combination of ROS production and SLC7A11 modulation played a key role in the over-expression of P-gp.

## Discussion

Although it is commonly recognized that redox signals modulate the transporters of membrane proteins at genetic and proteomic levels^7–14^, the underlying mechanism that contributes to P-gp over-expression remain unclear. Current evidence has shown that the environmental scavenging of ROS (NAC) and the modulation of endogenous GSH synthesis affected the expression and function of P-gp similarly^32, 33^. In our present research, the combinational effects of ROS addition and the partial silencing of SLC7A11 greatly promoted the expression and activity of P-gp in MCF-7 cells (Fig. 6). By contrast, the single factor of either added ROS or down-regulated SLC7A11/inhibited influx of cystine only increased P-gp expression several times. Interestingly, NAC could also reverse the up-regulation of P-gp induced by SLC7A11 or cystine (Figs 3–5). Indeed, our findings strongly indicated that ROS and SLC7A11 were two related factors responsible for the expression and function of P-gp, and the combinational effect of increased ROS production and reduced ability of ROS scavenging (i.e., inhibition of SLC7A11 and reduced GSH synthesis) played a key role in the over-expression of P-gp induced by ADR. Recently, X Tang *et al*. reported that cystine deprivation triggered necrosis in triple-negative breast cancer (TNBC) cells^34^, and Feng Wang *et al*. found that ADR increased the expression of SLC7A11 in TNBC, while the inhibition of the SLC7A11 antiporter system sensitized TNBC cells to ADR^35^. These results indicated that SLC7A11 was a potential target for the enhancement of the anticancer efficacy of conventional therapy in patients with TNBC. However, our study clearly showed that ADR inhibited cystine influx, down-regulated SLC7A11 transporter activity in MCF-7 cells, and efficiently increased P-gp expression and function. The differential effects of ADR were probably dependent on the diverse subtypes of breast cancer cells, i.e., MCF-7 cells are luminal-type cells, while TNBC cells are basal-type cells^34^.

**Figure 7:**
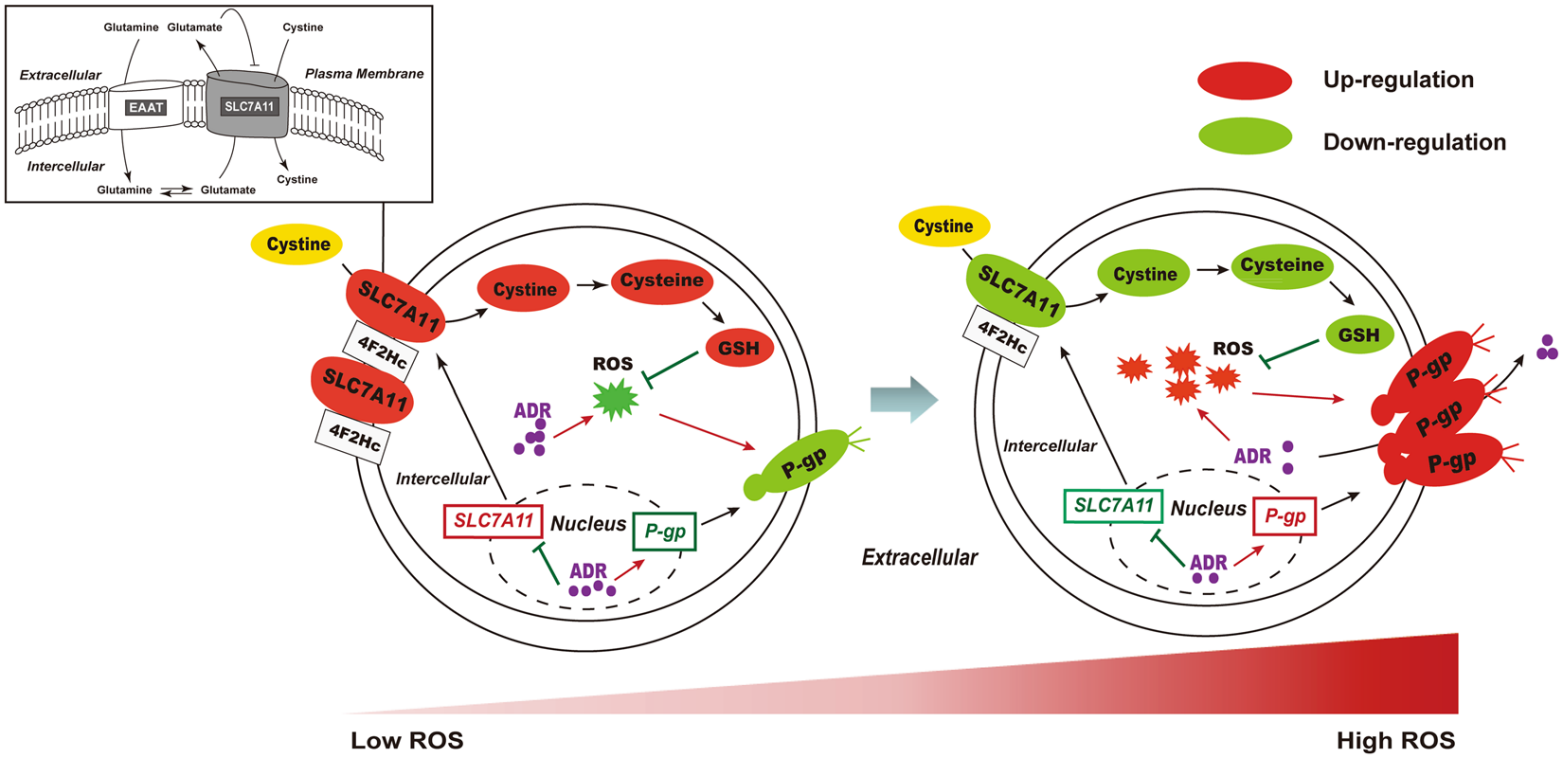
A schematic diagram of the reprogrammed homeostasis of GSH synthesis and subsequent P-gp expression of MCF-7S into MCF-7R cells, where ROS, SLC7A11 and cystine play an important role in the process. Red indicates up-regulation and green indicates down-regulation.

Actually, the mechanism of multidrug resistance is primarily involved in the up-regulation of multiple resistant transporters and has been well documented at the transcriptional and posttranscriptional levels. Mechanistic studies have suggested that redox signals modulate the transporters of membrane proteins^36–38^. However, the underlying biochemical process remains largely unclear regarding how ADR perturbs redox homeostasis and the identity of the crucial factor that dominates the increased expression of P-gp. To explore the underlying mechanism that contributes to the over-expression of P-gp, it was necessary to conduct a series of analyses on MCF-7S cells. In addition, data from MCF-7R cells (with hundreds of times more P-gp expression) could provide more information to confirm the mechanism that was found in MCF-7S cells. For example, our previous and present study revealed that there was a significant difference between MCF-7S and MCF-7R cells in terms of metabolic pattern^20^, intracellular ROS level, intracellular GSH/GSSG ratio, SLC7A11 expression and cystine uptake (Fig. 1). Thus, we believed that these differences were involved in multiple resistance. What’s more, data from MCF-7R cells assisted in providing strategies in modulating or reversing the over-expression of P-gp. For example, we observed that supplementary cystine could reduce the expression and function of P-gp, indicating its potential clinical value. However, to the best of our knowledge, no previous reports demonstrated a link between the SLC7A11/cystine metabolic pathway and the regulation of the efflux transporter P-gp. For the first time, we confirmed that the combination of elevated ROS and the inhibition of SLC7A11/cystine was the underlying mechanism that contributed to the over-expression of P-gp induced by ADR (Fig. 7). Furthermore, the SLC7A11 transporter may be a potential target to modulate resistance.

## Methods

### Cell lines and culture

Human breast cancer MCF-7 cells and the ADR-resistant MCF-7 subline (MCF-7R) were obtained from the Institute of Hematology and Blood Diseases Hospital (Tianjin, China). These cell lines were grown in RPMI-1640 medium supplemented with 10% (v/v) foetal bovine serum, 100 U/mL penicillin and 0.1 mg/mL streptomycin (Invitrogen, Carlsbad, CA, USA) at 37 °C in a 5% CO2 incubator.

### Intracellular cystine and glutamic acid measurement

Cystine and glutamic acid were identified by metabolomic analysis as reported previously^20^ and were compared with their respective reference standards. A relative quantitative measurement of intensity was achieved by calculating the area under the curve (AUC) of each peak, with a quant mass of m/z 348 and a retention time of 9.46 min for glutamic acid, and a quant mass of m/z 411 and a retention time of 12.99 min for cystine.

### Intracellular glutathione and ROS Measurement

MCF-7S and MCF-7R cells were cultured in 12-well plates. After treatment, the cells were harvested, and then the total glutathione (GSH and GSSG) was measured using commercially available glutathione assay kits according to the manufacturer’s instructions (Beyotime, China). For ROS detection, cells were incubated for 30 min with 10 μM 2′,7′-dichloro-fluorescein diacetate (DCFDA), harvested and analysed in triplicate using a microplate reader Powerwave 200 (Bio-Tek Instruments, Winooski, VT, USA) at wavelength of 488/535 nm. The GSH, GSSG and ROS values were also normalized to the protein levels in each sample.

### Transient SLC7A11 siRNA transfection

Cells were plated in six-well plates and transfected with siRNAs: SLC7A11 siRNA (h) (sc-76933, Santa Cruz, USA) and Control siRNA-A (sc-37007, Santa Cruz, USA) using the Lipofectamine® RNAiMAX Transfection Reagent (13778150, Life Technologies, USA) and the Gibco™ Opti-MEM™ I Reduced Serum Media (31985-070, Life Technologies, USA) according to the manufacturer’s instructions. After incubation for 72 hours, the cells were harvested for further experiments. The expression of SLA7A11 was monitored by reverse transcription-polymerase chain reaction (RT-PCR) and Western blot analysis to confirm the efficiency of the siRNA knockdown.

### Stable Transfection of MCF-7 cells

The human SLC7A11-expressing and empty vectors were kindly provided by Dr. M. Conrad (Institute of Clinical Molecular Biology and Tumour Genetics, GSF-Research Centre, Germany). Transfection of the MCF-7 cell line with SLC7A11-expressing or empty vectors was performed as reported previously^39^. After 24 h, the cells were stably transfected with 2 mg of DNA using the FuGENE® HD Transfection Regent (Roche, Swiss) according to the manufacturer’s instructions. After 48–72 h, the culture medium was replaced with fresh medium containing puromycin for the selection of stable transfectants. RT-PCR and Western blot were both used to confirm the efficiency of SLC7A11 transfection.

### Reverse transcription PCR

Total RNA was isolated from MCF-7S and MCF-7R cells via the TRIzol method^40^ in accordance with the manufacturer’s instructions and was quantified with ultraviolet spectrophotometry. Complementary DNA was synthesized with the PrimeScript RT Reagent Kit Perfect Real Time (Takara Biomedicals). The following primer sets were used in this study: human P-gp, 5′-GCTGGGAAGATCGCTACTGA-3′ and 5′-GGTACCTGCAAACTCTGA-3′; human SLC7A11, 5′-GCTGGGCTGATTTATCTTCG-3′ and 5′-GAAAGCTGGGATGAACAGT-3′; human Actin 5′-GCGTGACATTAAGGAGAAG-3′ and 5′-GGGAAGGAAGGCTGG AAGA-3′. The reverse transcription reaction conditions were as follows: 37 °C for 15 min followed by 85 °C for 5 s. PCR was conducted using SYBR Premix Ex TaqTM II (Takara Biomedicals) according to the manufacturer’s instructions. The PCR conditions were as follows: 95 °C for 15 s followed by 72 °C for 30 s and 60 °C for 60 s (40 cycles). All of the samples were quantified using the comparative Ct method for the relative quantification of gene expression, normalized to actin.

### Western blotting analysis

Proteins (40 μg) in the membrane extracts or in the whole cell lysate were separated by 10% sodium dodecyl sulphate-polyacrylamide gel (SDS-PAGE) and transferred to polyvinylidene fluoride membranes (Bio-Rad, Hercules, CA, USA). After blocking with 5% non-fat milk, the membrane was incubated with the primary antibodies against SLC7A11 (1:400, ab60171, Abcam, USA), P-gp (1:1000, ab170904, Abcam, USA), ATPase Na^+^/K^+^ (1:10000, ab185210, Abcam, USA) overnight at 4 °C, followed by incubation with the appropriate horseradish peroxidase (HRP)-linked secondary antibodies for 1 h at 37 °C. The signals were detected with an enhanced chemiluminescence kit (Thermo Fisher Scientific, Waltham, MA, USA) and were captured with a ChemiDoc XRS^+^ System (Bio-Rad, Hercules, CA, USA). The intensity of bands was quantified by Image Lab statistical software (Bio-Rad Laboratories, Hercules, CA, USA).

### Intracellular accumulation of Rhodamine 123 (Rho 123) Assay

The intracellular fluorescence intensity of Rho 123, a classic fluorescence substrate of P-gp, was monitored for the evaluation of P-gp function^41^. Thus, a high degree of intracellular fluorescence of Rho 123 represents the poor efflux function of P-gp. Briefly, MCF-7 cells were plated in 24-well plates and then were incubated with Hank’s Balanced Salt Solution (HBSS; 37 °C, pH 7.4) containing Rho123 (5 μM) for 2 h. The accumulation was stopped by rinsing the cells with ice-cold HBSS. The cells were lysed, and the protein concentrations were measured via the Bradford method using the BCA protein assay kit (Beyotime, Jiangsu, China). In a parallel experiment, the intracellular accumulation of Rho123 was detected by fluorescence microscopy, or with a microplate reader Powerwave 200 (Bio-Tek Instruments, Winooski, VT, USA) with the excitation wavelength set at 485 nm and the emission wavelength set at 546 nm. Fluorescence values of Rho 123 were also normalized to the protein levels in each sample.

### Cytotoxicity assay (MTT assay)

MCF-7 cell viability was evaluated using the 3-(4,5-dimethythiazole-2-yl)-2,5-diphenyl-tetrazolium bromide (MTT) assay. Cells were seeded into a 96-well plate (5 × 10^3^ cells/well). Following the treatment, the cells were incubated with 20 µL (5 mg/mL) MTT for 4 hours at 37 °C. The medium was removed and the formazan crystals produced by viable cells were dissolved in 150 µL DMSO for 10 min at 37 °C. The absorbance was detected using a microplate reader Powerwave 200 (Bio-Tek Instruments, Winooski, VT, USA) at a wavelength of 570/695 nm.

### Immunofluorescence assay of P-gp expression

MCF-7 cells were plated in 6-well plates before harvesting. After treatment and washing, the cells were fixed with 4% paraformal-dehyde solution, followed by washing and blocking. Next, the cells were incubated with the FITC-conjugated anti-P-gp polyclonal antibody or an isotype-matched negative control for 1 h at 37 °C (BD Biosciences, Franklin Lakes, NJ, USA). After washing, the cells were analysed via flow cytometry and the data were acquired on a BD FACSVerse (FACSCalibur, BD, Franklin Lakes, NJ, USA) and then were analysed with CELLQUEST software. MCF-7 cells were plated in Cell Imaging Coverglass (Eppendorf, USA), after treatment and washing, incubated with the FITC-conjugated anti-P-gp polyclonal antibody overnight at 4 °C and then laser confocal scanning microscopy analysis was performed (Live 5, Carl Zeiss, Oberkochen, Germany).

### Statistical analysis

All of the data were presented as the means ± SD. Significant differences between two groups were evaluated via Student’s t-test. All statistical analyses were performed with GraphPad Pro. Prism5.0 (GraphPad, San Diego, CA, USA). *p*-values < 0.05 was considered statistically significant. Data are representative of at least three independent experiments.

## Electronic supplementary material

Supplementary information

## References

[1] Stewart SL, King JB, Thompson TD, Friedman C, Wingo PA (2004). Cancer mortality surveillance-United States, 1990–2000. MMWR Surveill Summ..

[2] O’Driscoll L, Clynes M (2006). Biomarkers and multiple drug resistance in breast cancer. Curr Cancer Drug Targets..

[3] Gewirtz D (1999). A critical evaluation of the mechanisms of action proposed for the antitumor effects of the anthracycline antibiotics adriamycin and daunorubicin. Biochem Pharmacol..

[4] Puglisi F, Cardoso F, Lebrun F, Piccart DM (2006). First-Line Treatment of Metastatic Breast Cancer. Am J Cancer..

[5] Furusawa S (2001). Mechanism of resistance to oxidative stress in doxorubicin resistant cells. Biol Pharm Bull..

[6] Amirzada MI (2014). Recombinant human interleukin 24 reverses Adriamycin resistance in a human breast cancer cell line. Pharmacol Rep..

[7] Elliott AM, Al-Hajj MA (2009). ABCB8 mediates doxorubicin resistance in melanoma cells by protecting the mitochondrial genome. Mol Cancer Res..

[8] Takara K, Sakaeda T, Okumura K (2006). An update on overcoming MDR1-mediated multidrug resistance in cancer chemotherapy. Curr Pharm Des..

[9] Zhang F (2009). Anxa2 plays a critical role in enhanced invasiveness of the multidrug resistant human breast cancer cells. J Proteome Res..

[10] de Gooijer MC (2015). P-glycoprotein and breast cancer resistance protein restrict the brain penetration of the CDK4/6 inhibitor palbociclib. Invest New Drugs..

[11] Kovalchuk O (2008). Involvement of microRNA-451 in resistance of the MCF-7 breast cancer cells to chemotherapeutic drug doxorubicin. Mol Cancer Ther..

[12] Chekhun VF, Lukyanova NY, Kovalchuk O, Tryndyak VP, Pogribny IP (2007). Epigenetic profiling of multidrug-resistant human MCF-7 breast adenocarcinoma cells reveals novel hyper- and hypomethylated targets. Mol Cancer Ther..

[13] Villeneuve DJ (2006). cDNA microarray analysis of isogenic paclitaxel- and doxorubicin-resistant breast tumor cell lines reveals distinct drug-specific genetic signatures of resistance. Breast Cancer Res Treat..

[14] Smith L (2006). The analysis of doxorubicin resistance in human breast cancer cells using antibody microarrays. Mol Cancer Ther..

[15] Kuo MT (2009). Redox regulation of multidrug resistance in cancer chemotherapy: molecular mechanisms and therapeutic opportunities. Antioxid Redox Sign..

[16] Ouchi J, Ryu SY, Jhun BS, Hurst S, Sheu SS (2014). Mitochondrial ion channels/transporters as sensors and regulators of cellular redox signaling. Antioxid Redox Sign..

[17] Trivedi M, Shah J, Hodgson N, Byun HM, Deth R (2014). Morphine induces redox-based changes in global DNA methylation and retrotransposon transcription by inhibition of excitatory amino acid transporter type 3-mediated cysteine uptake. Mol Pharmacol..

[18] Ishii T, Mann GE (2014). Redox status in mammalian cells and stem cells during culture *in vitro*: Critical roles of Nrf2 and cystine transporter activity in themaintenance of redox balance. Redox Biology..

[19] Nakanishi K (2014). Persistent epicardial adipose tissue accumulation is associated with coronary plaque vulnerability and future acute coronary syndrome in non-obese subjects with coronary artery disease. Atherosclerosis..

[20] Cao B (2013). Metabolomic approach to evaluating adriamycin pharmacodynamics and resistance in breast cancer cells. Metabolomics..

[21] Sha LK (2015). Loss of Nrf2 in bone marrow derived macrophages (BMDMΦ) impairs antigen-driven CD8+ T cell function by limiting GSH and Cys availability. Free Radic Biol Med..

[22] Tsujita T (2014). Transcription factor Nrf1 negatively regulates the cystine/glutamate transporter and lipid-metabolizing enzymes. Mol Cell Biol..

[23] Ohly, Sara H (2012). The oxidative stress-inducible cystine/glutamate antiporter, system x (c) (−): cystine supplier and beyond. Amino Acids..

[24] Jiang L (2015). Ferroptosis as a p53-mediated activity during tumour suppression. Nature..

[25] Duan R (2012). Biphasic regulation of P-glycoprotein function and expression by NO donors in Caco-2 cells. Acta Pharmacol Sin..

[26] Wang SF (2013). 7-Ketocholesterol induces P-glycoprotein through PI3K/mTOR signaling in hepatoma cells. Biochem Pharmacol..

[27] Wartenberg M (2010). Glycolytic pyruvate regulates P-Glycoprotein expression in multicellular tumor spheroids via modulation of the intracellular redox state. J Cell Biochem..

[28] Flohé L (2012). The fairytale of the GSSG/GSH redox potential. Biochim Biophys Acta.

[29] Giustarini D, Dalle-Donne I, Milzani A, Fanti P, Rossi R (2013). Analysis of GSH and GSSG after derivatization with N-ethylmaleimide. Nature Protocol..

[30] Timmerman LA (2013). Glutamine Sensitivity Analysis Identifies the xCT Antiporter as a Common Triple-Negative Breast Tumor Therapeutic Target. Cancer Cells..

[31] Drayton RM (2014). Reduced expression of miRNA-27a modulates cisplatin resistance in bladder cancer by targeting the cystine/glutamate exchanger SLC7A11. Clin Cancer Res..

[32] Wu YM (2016). Inverse agonist of estrogen-related receptor α suppresses the growth of triple negative breast cancer cells through ROS generation and interaction with multiple cell signaling pathways. Oncotarget..

[33] Bauzo RM, Kimmel HL, Howell LL (2012). The cystine–glutamate transporter enhancer N-acetyl- l -cysteine attenuates cocaine-induced changes in striatal dopamine but not self-administration in squirrel monkeys. Pharmacol Biochem Behav..

[34] Tang, X. *et al*. Cystine addiction of triple-negative breast cancer associated with EMT augmented death signaling. *Oncogene* (2016). 10.1038/onc.2016.394 PMC5438912 27869167

[35] Wang F, Yang Y (2015). Retraction Note to: Suppression of the xCT–CD44v antiporter system sensitizes triple-negative breast cancer cells to doxorubicin. Breast Cancer Res Treat..

[36] Daflonyunes N (2013). Characterization of a multidrug-resistant chronic myeloid leukemia cell line presenting multiple resistance mechanisms. Mol Cell Biochem..

[37] Wang H (2014). Multiple mechanisms underlying acquired resistance to taxanes in selected docetaxel-resistant MCF-7 breast cancer cells. BMC Cancer..

[38] Zhang Y (2015). Cbl-b inhibits P-gp transporter function by preventing its translocation into caveolae in multiple drug-resistant gastric and breast cancers. Oncotarget..

[39] Banjac A (2008). The cystine/cysteine cycle: a redox cycle regulating susceptibility versus resistance to cell death. Oncogene..

[40] Mannhalter C, Koizar D, Mitterbauer G (2000). Evaluation of RNA isolation methods and reference genes for RT-PCR analyses of rare target RNA. Clinical Chemistry & Laboratory Medicine Cclm..

[41] Liu J (2015). Chronic inflammation up-regulates P-gp in peripheral mononuclear blood cells via the STAT3/Nf-κb pathway in 2,4,6-trinitrobenzene sulfonic acid-induced colitis mice. Sci Rep..

