# Supplementary material for: The down-regulation of SLC7A11 enhances ROS induced P-gp over-expression and drug resistance in MCF-7 breast cancer cells
Source: Sci Rep. 2017 Jun 19;7:3791. doi: 10.1038/s41598-017-03881-9 (PMC5476638; doi:10.1038/s41598-017-03881-9)
Supplement: Supplementary file 1 — Supplementary information [file 41598_2017_3881_MOESM1_ESM.pdf]

# Supplementary Information

# The down-regulation of SLC7A11 enhances ROS induced P-gp over-expression and drug resistance in MCF-7 breast cancer cells

Chun Ge[1,†], Bei Cao[2,†], Dong Feng[1], Fang Zhou[1], Jingwei Zhang[1], Na Yang[1], Siqi Feng[1], Guangji Wang[1,*], Jiye Aa[1,*]

[†]These authors contributed equally to this work.

[1] Laboratory of Metabolomics, Key Laboratory of Drug Metabolism and Pharmacokinetics, China Pharmaceutical University, Nanjing 210009, CHINA;

[2] Nanjing Drum Tower Hospital, the Affiliated Hospital of Nanjing University Medical School, Nanjing 210009, CHINA

***** To whom correspondence should be addressed: Professor Jiye Aa or Professor Guangji Wang, China Pharmaceutical University, Tongjiaxiang 24, Nanjing 210009, CHINA.

* Correspondent email: jiyea@cpu.edu.cn or guangjiwang@hotmail.com

## Supplementary figures

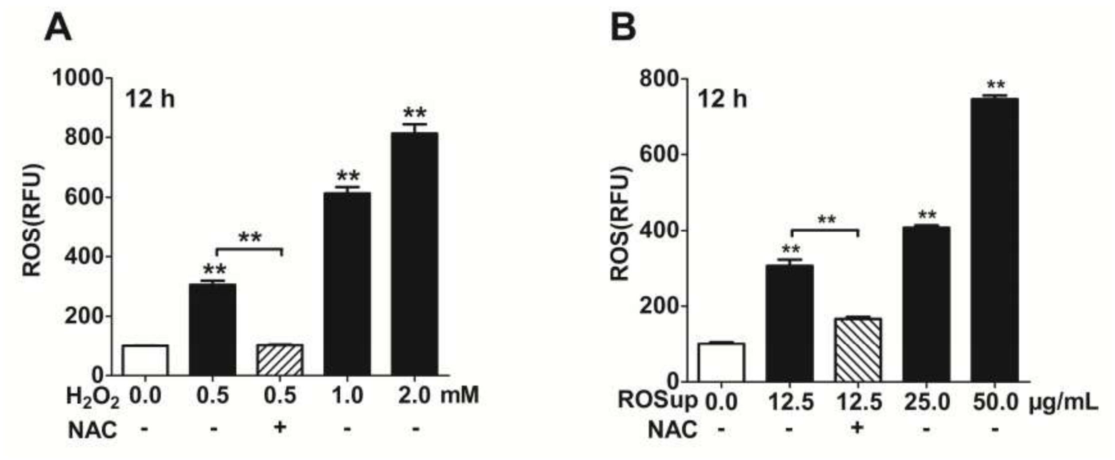


**Supplementary Figure S1. Effects of ROS agents on ROS generation in MCF-7S cells.** (**A**) $H_2O_2$ increased intracellular ROS generation, which could be reversed by NAC (10 mM, 30 min of pretreatment). (**B**) ROSup increased intracellular ROS generation, which could be reversed by NAC (10 mM, 30 min of pretreatment).

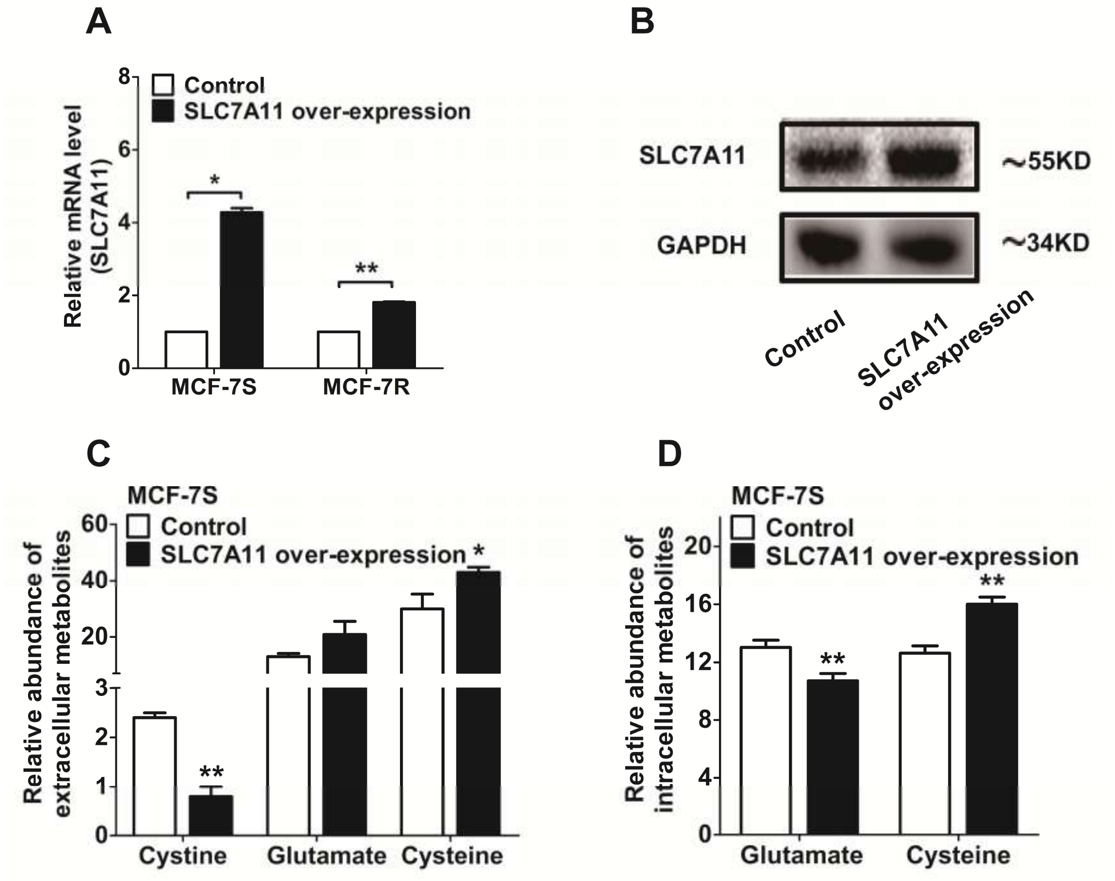


**Supplementary Figure S2. Validation at the gene, protein and function levels in SLC7A11 over-expressed MCF-7S and MCF-7R cells.** (**A**) SLC7A11 mRNA expression was increased in SLC7A11 over-expressed MCF-7S and MCF-7R cells. (**B**) SLC7A11 protein expression was increased in SLC7A11 over-expressed MCF-7S cells. (**C-D**) Influx of cystine, efflux of glutamate and cysteine production were enhanced in SLC7A11 over-expressed MCF-7S cells.
